# Dynamic Characteristics of Ventilatory and Gas Exchange during Sinusoidal Walking in Humans

**DOI:** 10.1371/journal.pone.0168517

**Published:** 2017-01-11

**Authors:** Yoshiyuki Fukuoka, Masaaki Iihoshi, Juhelee Tuba Nazunin, Daijiro Abe, Yoshiyuki Fukuba

**Affiliations:** 1 Laboratory of Environmental Physiology, Faculty of Environmental and Symbiotic Sciences, Prefectural University of Kumamoto, Kumamoto, Japan; 2 Laboratory of Environmental Physiology, Faculty of Health and Sports Science, Doshisha University, Kyotanabe, Japan; 3 Biodynamics Laboratory, Center for Health and Sports Science, Kyushu Sangyo University, Fukuoka, Japan; 4 Department of Sports Science and Physiology, Hiroshima Prefectural University, Hiroshima, Japan; Universita degli Studi di Verona, ITALY

## Abstract

Our present study investigated whether the ventilatory and gas exchange responses show different dynamics in response to sinusoidal change in cycle work rate or walking speed even if the metabolic demand was equivalent in both types of exercise. Locomotive parameters (stride length and step frequency), breath-by-breath ventilation (*V̇*_E_) and gas exchange (CO_2_ output (*V̇*CO_2_) and O_2_ uptake (*V̇*O_2_)) responses were measured in 10 healthy young participants. The speed of the treadmill was sinusoidally changed between 3 km·h^-1^ and 6 km·h^-1^ with various periods (from 10 to 1 min). The amplitude of locomotive parameters against sinusoidal variation showed a constant gain with a small phase shift, being independent of the oscillation periods. In marked contrast, when the periods of the speed oscillations were shortened, the amplitude of *V̇*_E_ decreased sharply whereas the phase shift of *V̇*_E_ increased. In comparing walking and cycling at the equivalent metabolic demand, the amplitude of *V̇*_E_ during sinusoidal walking (SW) was significantly greater than that during sinusoidal cycling (SC), and the phase shift became smaller. The steeper slope of linear regression for the *V̇*_E_ amplitude ratio to *V̇*CO_2_ amplitude ratio was observed during SW than SC. These findings suggested that the greater amplitude and smaller phase shift of ventilatory dynamics were not equivalent between SW and SC even if the metabolic demand was equivalent between both exercises. Such phenomenon would be derived from central command in proportion to locomotor muscle recruitment (feedforward) and muscle afferent feedback.

## Introduction

Walking and cycling are two of the most popular endurance exercises that can be performed at one’s own pace. A complex ventilatory and/or gas exchange response during such endurance exercises can be simply described by phase shift (*PS*) and amplitude (*A*) when exercise intensity is changed sinusoidally [1,2]. The *PS* and *A* are the index of the speed of ventilatory and gas exchange dynamics and its response characteristics. A combination of these parameters could have differentiated athlete’s aerobic fitness without maximal effort by participants [3]. During dynamic exercise on a cycle ergometer with sinusoidal work rate changes, the *A* of ventilation (*V̇*_E_) response decreases while the *PS* of the *V̇*_E_ response increases when the work rate oscillation periods were shortened [4–7]. Considering the control mechanisms of the ventilatory response during human walking, a widely accepted hypothesis is proposed that the supra-spinal sites, which may control the spinal pattern generators for locomotion, stimulate the breathing [8,9]. More specifically, we sought to establish whether a temporal relationship exists between the *V̇*_E_ and locomotor movement during walking, because the major locomotor variables, such as stride length and step frequency, had a significant stimulation on the respiratory center via the supra-spinal sites [8–10]. Exercise hyperpnea should be more facilitated during walking than cycling, because additional mechanical stimulus induces by walking via the supra-spinal locomotor center to the respiratory center [11–13]. It is hypothesized that walking has the additional greater ventilatory responses compared with cycling even when the metabolic demand is equivalent.

With regard to a step-transition of work rate or speed change during cycling or walking, the speed of O_2_ uptake (*V̇*O_2_) dynamics was not significantly different at moderate and severe intensities [14]. However, this comparison was not carried out under the equivalent metabolic demand, because the muscle recruitment pattern must be different between walking and cycling. This is because cycling causes greater intramuscular tension development, resulting in an increase in fast-twitch fiber recruitment [11]. In contrast, an increase in the slow-twitch fiber recruitment was strongly related to the speed of the O_2_ uptake (*V̇*O_2_) dynamics or oxidative capacity [3,7,15]. The results of previous studies bring our second hypothesis that the *V̇*O_2_ dynamics will be faster during walking with sinusoidal speed changes (SW) than cycling with sinusoidal work rate changes (SC) due to different muscle fiber recruitment even at the equivalent metabolic demand. To test these two hypotheses, our present study investigated whether the ventilatory and gas exchange responses showed different dynamics in response to sinusoidal work rate or speed changes in both types of exercise at the equivalent metabolic demand.

## Materials and Methods

### Subjects

The subjects of the present study were 10 healthy young men and women volunteers (men = 5, women = 5; age: 21.2 ± 0.6 yrs; height: 166.1 ± 1.6 cm; weight: 59.0 ± 2.4 kg; mean ± SE), none of whom was on medication known to affect cardiovascular function. The subjects were fully informed of any risks and discomforts associated with these experiments before giving their written, informed consent to participate in the present study, which was approved by the ethics committees of the Institutional Review Board of Prefectural University of Kumamoto and Doshisha University.

### Measurements

A mass-flow sensor (type AB, Minato Medical Sciences, Japan) was fit to the expiratory port of the valve to continuously record expiratory airflow, which was calibrated before each measurement with a 3-liter syringe at three different flow rates. Tidal volume (VT) and *V̇*_E_ were calculated by integrating the flow tracings recorded at the mouth of the subject. We confirmed that the sensitivity of the hot wire anemometer did not alter with changes in gas composition over the range of physiological flow variations. Expiratory PO_2_ and PCO_2_ were determined by mass spectrometry (WSMR-1400, Westron, Japan) from a sample drawn continuously from the inside of the mouthpiece at 1 ml/s; the loss of volume, however, was neglected in our calculations. Two reference gases of known concentrations (O_2_ 15.04%, CO_2_ 2.92%, and N_2_ 82.04%; O_2_ 11.93%, CO_2_ 6.96%, and N_2_ 81.11%) and room air were used to calibrate the mass spectrometer. The volumes, flows, PCO_2_ and PO_2_ at the mouth were recorded in real time with a 50-Hz sampling frequency using a computerized on-line breath-by-breath system (AE-280, Minato Medical Sciences, Japan) from time aligned gas volume and concentration signals. Breath-by-breath *V̇*_E_ (BTPS), *V̇*O_2_ (STPD), and *V̇*CO_2_ (STPD), the respiratory exchange ratio (R), and end-tidal PCO_2_, and PO_2_ (P_ET_CO_2_, P_ET_O_2_) were determined. Heart rate (HR) was measured by beat-to-beat counting from the *R* spike of the ECG.

The signal controlling the speed of the motor driving the treadmill (modified TMS 2200, Nihon Koden, Japan) was delivered by a microcomputer through a digital–analog converter. Also, the electromagnetically braked ergometer (RS-232c Combi, Japan) was also controlled by a microcomputer through RS-232C.

### Protocol

Two questions needed to be clarified prior to the present study: (1) how long time delay was needed between food ingestion and the tests needed to reduce the effects of eructation, which interfere with the breath-by-breath ventilatory measurements; (2) how much time was needed to reach a steady state before starting the sinusoidal exercise.

**Fig 1:**
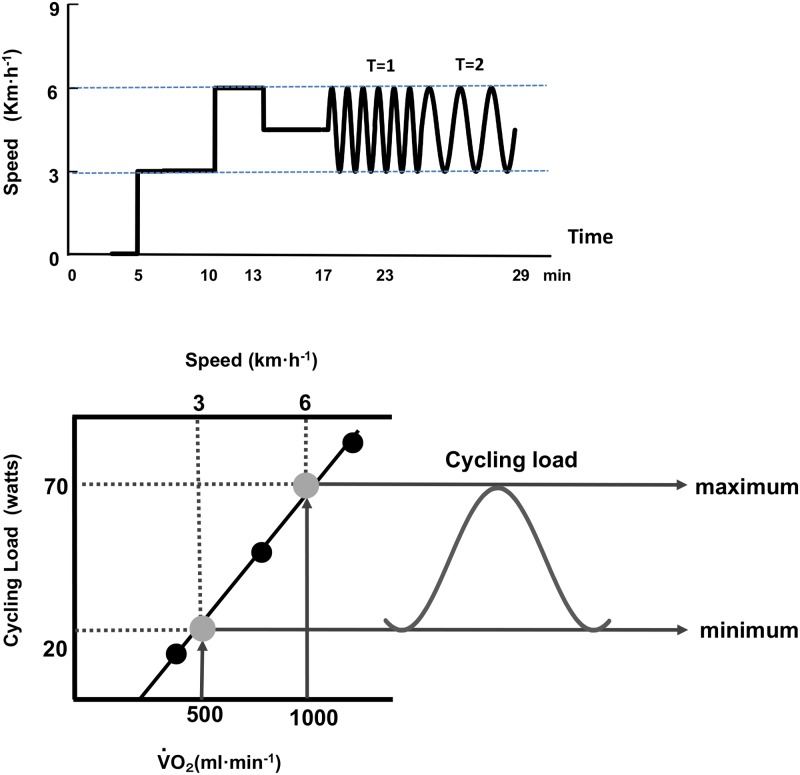
Scheme for setting work rate at the equivalent metabolic demand between walking and cycling. First, the mean values of *V̇*O_2_ at two constant treadmill speed of 3 km·h^-1^ and 6 km·h^-1^ (upper panel) should be obtained. Second, the regression line between work rate of cycling and *V̇*O_2_ was calculated, we selected three step work rates of cycling between 20 to 100 watts. At the speeds of 3 km·h^-1^ and 6 km·h^-1^, corresponding work rates were calculated from this regression line (lower panel).

First, we sought to obtain the mean values of *V̇*O_2_, *V̇*CO_2_ at two constant treadmill speeds of 3 km·h^-1^ and 6 km·h^-1^. Second, we measured the mean values of *V̇*O_2_, *V̇*CO_2_ at selected three adequate work rates of cycling between 20 and 100 watts (i.e., 20, 50, and 80 watts in females and 40, 70, and 100 watts in males) in order to estimate the regression line between the work rate of cycling and *V̇*O_2_. At the speeds of 3 and 6 km·h^-1^, *V̇*O_2_ reached approximately 550 ml·min^-1^ and 1000 ml·min^-1^, corresponding to average work rates of 21.4 ± 2.3 and 67.8 ± 4.4 watts, respectively, these work rates of cycling were calculated from this regression line (Fig 1).

Second, following 5 min of walking at constant speed at the midpoint of the sinusoidal walking speed (4.5 km·h^-1^), the treadmill speed was changed with a sinusoidal pattern from 3 km·h^-1^ to 6 km·h^-1^ at a period (*T*) of 10, 5, 2 and 1 min and in a stepwise manner (steady-state) at the speeds of 3 km·h^-1^ and 6 km·h^-1^ for each 5 min [4,16–18]. The sinusoidal loading was repeated for five cycles at 1 min periods and continued three cycles at 2 min periods following warming up at a constant work load at the midpoint between the maximum and minimum. On another day, another sinusoidal loading was also repeated for three cycles at 5 min periods and continued two cycles at 10 min periods following 5 min warming up. Each sinusoidal period of oscillation was studied on a separate occasion (one session at a time, two sessions per week for each individual). The subjects walked freely on the treadmill. The SC measurements were carried out in the same manner, i.e., *T* and stepwise manner, while controlling the metabolic demand is the same as SW. The frequency of cycling remained constant at 60 revolutions per minute when the subjects performed SC.

In four out of ten subjects, locomotive step frequency and stride length were measured with a switch activated by stepping on a sensor on the sole of the right foot in each protocol. The signals from the treadmill and the stepping sensor were fed into a data acquisition system (PowerLab system, A/D Instruments, Castle Hill, Australia) and temporally aligned to the ventilatory data.

### Data analysis

All the data were analyzed using Fourier analysis as previously reported [2–7,16–19]. The variation in the treadmill speed or work rate was regarded as the input function. The *A* (i.e., mean to peak) and the *PS* of the fundamental component (same frequency as the input function) of the *V̇*_E_, *V̇*O_2_, *V̇*CO_2_, and end tidal PCO_2_ (P_ET_CO_2_) responses as well as the locomotion responses (locomotive step frequency and stride) were computed as follows:

$$A={\left({\text{Re}}^{2}+{\text{Im}}^{2}\right)}^{0.5}$$ (1)

$$PS={\text{tan}}^{-1^{}}(\text{Re}/\text{Im})$$ (2)

Where Re and Im are the real and imaginary parts of the response determined after second-by-second interpolation of the respiratory and locomotor (*x*) responses as:

$$\text{Re}=\frac{2}{NT}\sum_{t=0}^{NT}\left(x(t)-Mx)\text{cos}(2\pi ft\right)$$ (3)

and

$$\text{Im}=\frac{2}{NT}\sum_{t=0}^{NT}\left(x(t)-Mx)\text{sin}(2\pi ft\right)$$ (4)

Where *x* (*t*) is the response value at time *t* (in seconds), *M*x is the mean value of *x* for an integer number of cycles (*N*), *T* is the period of the input signal (in seconds) and *f* (= 1 / *T*) is its frequency in cycles per second.

The ratios of *A* of the respiratory and locomotive parameters against sinusoidal change in work rate or walking speed were normalized by dividing the magnitude of parameters from 3 to 6 km·h^-1^ or from 21.4 ± 2.3 to 67.8 ± 4.4 watts during each steady-state exercise, and those were presented as *A* ratio (%). Even though we established the equivalent metabolic demand (*V̇*O_2_ and *V̇*CO_2_), we thought that the *A* ratio of *V̇*_E_ dynamics could be characterized the specific dynamics of ventilation during SW or SC.

### Statistical analysis

All values are presented as means ± standard error (x̄ ± SE), which represents a within-participant deviation to estimate a range for the “true” mean value. The significance of difference for each variable (*V̇*_E_, *V̇*O_2_, *V̇*CO_2_, and P_ET_CO_2_) was determined by two-way analysis of variance (ANOVA) comparing exercise mode (cycling and walking) × oscillation frequency period (*T*; 1 min– 10 min) and by one-way analysis of variance (ANOVA) comparing oscillation frequency period for step frequency and stride length. *Tukey’s* test was applied for the appropriate data sets if a significant *F* value was obtained. The value of *H* (after correction for similar values) and the corresponding *P* values are given in the text for each variable. The level of significance was set at p < 0.05.

## Results

**Fig 2:**
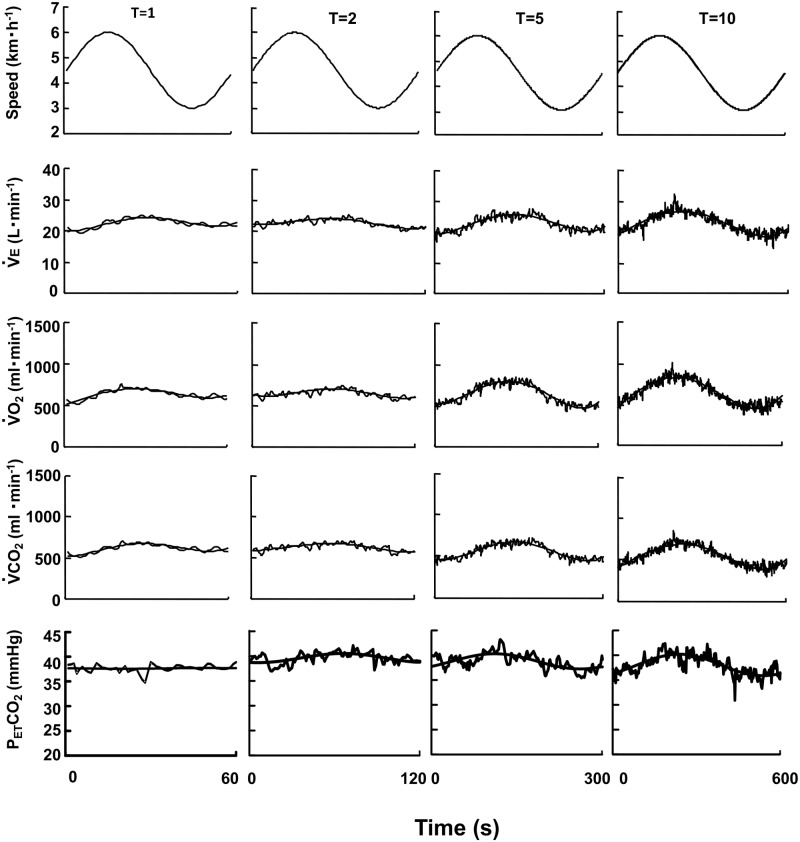
Time course of the ventilatory and gas exchange responses at different oscillation periods. Example in a representative subject of the ventilatory and gas exchange variables of ventilation (*V̇*_E_), O_2_ uptake (*V̇*O_2_), CO_2_ output (*V̇*CO_2_), and end-tidal PCO_2_ (P_ET_CO_2_) responses against four different oscillation periods during SW at *T* = 1, 2, 5, and 10 min. Oscillating line is the superimposed gas exchange variables data. Smooth line is the sine-wave fundamental component of these dynamics.

Fig 2 displays representative data of the ventilatory and gas exchange dynamics and the calculated fundamental components during SW across all measured periods. The mean values of all ventilatory and gas exchange dynamics showed no significant differences between SW and SC (Table 1). The *A* and *PS* could be reliable variables to estimate the fundamental component of the ventilatory and gas exchange dynamics during SW and SC at the equivalent metabolic demand.

**Table 1:** Mean value of gas exchange and locomotive variables during sinusoidal walking and sinusoidal cycling.

	walking	cycling
VO_2_ (ml min^-1^)	743 ± 33	747 ± 45
VCO_2_ (ml min^-1^)	716 ± 46	711 ± 39
V_E_ (L min^-1^)	22.23 ± 1.02	21.96 ± 1.04
HR (beats min^-1^)	101 ± 3	98 ± 3
R	0.96 ± 0.01	0.95 ± 0.01
P_ET_CO_2_ (mmHg)	40.0 ± 3.9	39.1 ± 3.8
stride length (cm)	69.1 ± 0.80	
step frequency (steps min^-1^)	109.0 ± 0.3	

Breath-by-breath ventilation (V_E_, BTPS), O_2_ uptake (VO_2_, STPD), CO_2_ output (VCO_2_, STPD), the respiratory exchange ratio (R), heart rate (HR), and end-tidal PCO_2_ (P_ET_CO_2_) were determined. Data are shown by mean ± SE.

### Ventilatory and gas exchange dynamics between cycling and walking

**Fig 3:**
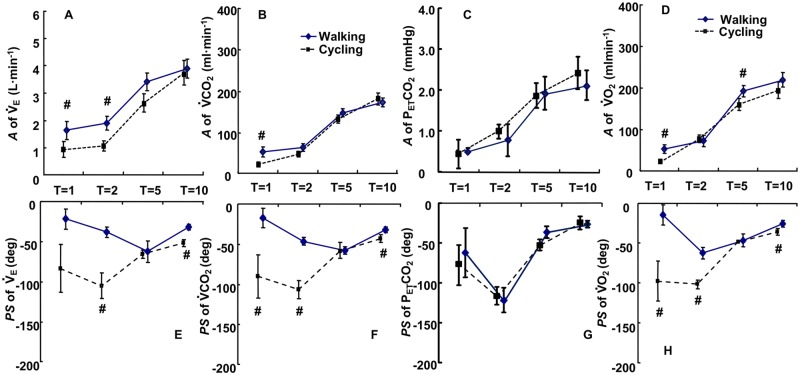
Comparisons of *A* and *PS* of ventilatory and gas exchange dynamics between SW and SC. Comparisons of *A* and *PS* between SW (solid line) and SC (dotted line) as a function of the periods of the sinusoidal changes in the treadmill speed and the cycling work rate at the equivalent metabolic demand. #,##; p < 0.05, 0.01 *vs*. cycling. Data are shown in mean ± SE.

During SW, the *A* of the *V̇*_E_ response decreased from 3.91 ± 0.30 L·min^−1^ (during constant speed exercise) to 1.70 ± 0.20 L·min^−1^ as *T* was shortened (Fig 3A). It was significantly greater in the periods of 1 min and 2 min during SW compared to SC (p < 0.05). The *A* of *V̇*CO_2_ decreased from 173 ± 10 ml·min^−1^ (*T*: 10 min) to 57 ± 9 ml·min^−1^ (*T*: 1 min) without a significant difference between SW and SC from 2 min to 10 min expect 1 min period (Fig 3B). The changes in P_ET_CO_2_ during SW displayed relatively smaller *A* oscillations ranging from 0.5 ± 0.1 to 2.1 ± 0.7 mmHg with a stable mean P_ET_CO_2_ varying from 39.3 ± 3.5 to 40.6 ± 3.7 mmHg as a whole (Fig 3C). The *A* of the *V̇*O_2_ response during SW decreased from 220 ± 17 ml·min^−1^ (*T*: 10 min) to 58 ± 11 ml·min^−1^ (*T*: 1 min). It was significantly greater during 1 min and 5 min periods compared to SC, in contrast, there were no significant differences in the periods of 2 min and 10 min (Fig 3D).

The *PS* of the *V̇*_E_ response during SW was significantly lesser compared to that during SC at the periods of 2 min (SW;—38.1 ± 6.6 deg, SC;—105.0 ± 15.0 deg) and 10 min (SW;—31.9 ± 3.3 deg, SC;—51.5 ± 4.2 deg) (p < 0.05). Thus, the ventilatory responses during SW were remarkably accelerated in comparison to those during SC (Fig 3E). Similarly, the *PS* of the *V̇*CO_2_ response became gradually greater at shorter periods during SW, and it became significantly smaller than that those observed during SC at the periods of 1, 2, and 10 min except for the period of 5 min (Fig 3F). The *PS* of the P_ET_CO_2_ response was not significantly different between SW and SC at any periods (Fig 3G, *F* = 0.89). The *PS* of the *V̇*O_2_ response gradually increased at shorter periods during SW, and it became significantly smaller than those observed during SC at the periods of 1, 2, and 10 min except for the period of 5 min (Fig 3H).

### Dynamics for locomotive parameters during sinusoidal walking

**Fig 4:**
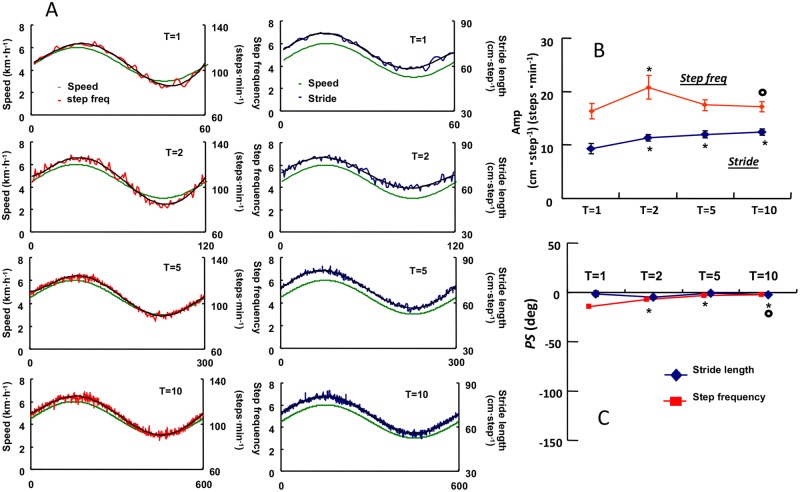
Time course of the locomotive responses at different time periods. Example of a representative subject of the locomotion pattern divided between the step frequency (left panels) and the stride length (right panels) during SW for all periods from 1 min to 10 min (sinusoidal speed change was from 3 km·h^*−*1^ to 6 km·h^*−*1^). Oscillating line is the superimposed step frequency and stride length. Smooth line is the sine-wave fundamental component of the dynamics (A). The *A* of the step frequency and stride length remained almost unchanged (B). The *PS* for the step frequency and stride length were quite small and virtually close to zero at each period (C). * p < 0.05 *vs*. T:1min, ○ p < 0.05 *vs*. T:2min. Data are shown in mean ± SE.

The step frequency and stride length followed the sinusoidal changes in treadmill speed (Fig 4A). Even though the specific characteristics of the *A* of ventilatory and gas exchange dynamics became gradually lesser during both exercises when the period was shortened, we did not find any trends in common in the *A* of step frequency with the statistically significant differences between periods of 1 min and 2 min and between 2 min and 10 min (p < 0.05) (Fig 4B). However, the *A* of stride length slightly increased as the period of SW increased, even though the significant differences in stride length were found between 1 min and other longer periods (p < 0.05).

In contrast, the *PS* between the changes in treadmill speed and locomotive parameters (stride length and step frequency) was very small at all periods, the largest *PS* was observed at the 1 min period in the step frequency, which was, at most,—14.5 ± 5.3 deg. When such a value was converted into time delay, it was only 2.4 ± 0.9 s. It demonstrated that the locomotive parameters have a quick response against sinusoidal speed change regardless of period length. However, a statistically significant decrease in the *PS* was found in the step frequency between 1 min and other longer periods, while the *PS* of the stride length remained unchanged even at the shortest period (Fig 4C).

### Accelerated V̇_E_ response during sinusoidally transient locomotion

**Fig 5:**
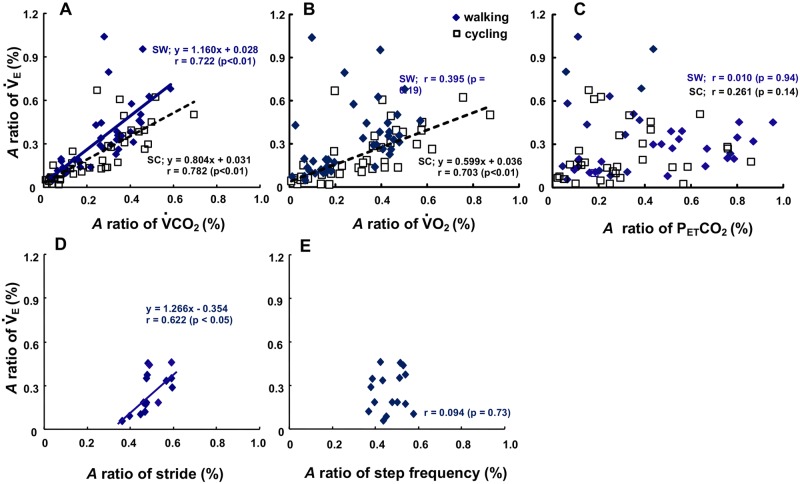
Relationship between the *A* ratio of locomotive and gas exchange parameters during SW and SC. The *A* ratio of the *V̇*_E_ against *V̇*O_2_ (A), *V̇*CO_2_ (B), and P_ET_CO_2_ (C) during SW and SC, respectively. Locomotive responses (step frequency (D) and stride length (E)) were presented. Note that the *A* ratio for the *V̇*_E_ correlated to the *A* ratio of the *V̇*CO_2_ closer during SW than SC. In contrast, the *A* ratio for *P*_ET_CO_2_ was not tightly related to the *V̇*_E_ dynamics. The slope of the regression lines of the *V̇*_E_-*V̇*CO_2_ relationship was steeper during SW than SC (p < 0.05). SL; y = 1.160x + 0.028, r = 0.722 (p < 0.01), SC; y = 0.804x + 0.031, r = 0.782 (p < 0.01).

The *A* ratio (*A* ratio for *V̇*_E_ between constant and sinusoidal load intensity variation) of the *V̇*_E_ response was closely related to the *A* ratio of *V̇*CO_2_ among the data obtained from all periods during SW and SC (SW; r = 0.722, SC; r = 0.782, p < 0.01) (Fig 5A). The slope of the regression lines of the *V̇*_E_-*V̇*CO_2_ relationship was steeper during SW (1.160, Fig 5A) than SC (0.804, Fig 5A), indicating that a 1.44 steeper slope was obtained during SW than SC.

In contrast, a significant correlation between the *A* ratio of the *V̇*O_2_ and *V̇*_E_ response was found during SC (Fig 5B; r = 0.703, p < 0.01), but not during SW (Fig 5B; r = 0.395, p = 0.19). Apparently, the ventilatory fluctuation is linked to the fluctuations in metabolism through CO_2_ production, but not through O_2_ consumption. The *A* ratio of the *V̇*_E_ was not significantly related to that of P_ET_CO_2_ during both exercises (Fig 5C). Although the *A* ratio of the *V̇*_E_ during SW was not significantly related to the changing step frequency of locomotion (r = 0.094, p = 0.73, Fig 5E), it was significantly related to the *A* ratio of the stride length (r = 0.622, p < 0.05, Fig 5D).

## Discussion

The frequency domain analyses of the *V̇*O_2_, *V̇*CO_2_, and *V̇*_E_ dynamics during SW and SC at the equivalent metabolic demand revealed that they were remarkably faster during walking (SW) than during cycling (SC) (Fig 3). Locomotion related parameters, such as step frequency and stride length, were rapidly adapted to the sinusoidal change in treadmill speed at any periods (Fig 4C). Therefore, the locomotor dynamics was mostly synchronized with sinusoidal changes in treadmill speed. A 1.44 times steeper *V̇*_E_-*V̇*CO_2_ slope was observed during SW, suggesting that the ventilatory responses were not equivalent between SW and SC even at same metabolic demand.

The physiological meanings of the *V̇*_E_ dynamics during either SC or SW have been investigated with the frequency domain analysis [4–6]. Thus, during mild sinusoidal exercise such as walking and cycling (average HR ≤ 100 beats·min^−1^), the *V̇*_E_ response is likely to be the most comparable with regard to the “Phase II” responses to step work rate change during cycle ergometer exercise [4,5,20–23]. In fact, regarding our results showing a tight association between *V̇*_E_ and *V̇*CO_2_ at the longer periods (5 min and 10 min), there would be no or minimal contribution to the ventilatory changes from the neural signals through the motor activity and/or central command, which would contribute to a faster *V̇*_E_ response to the step work rate initiated from the rest [10]. Haouzi *et al*. [16] also reported that parallel adjustments of the locomotor activity and *V̇*_E_ were observed during treadmill walking in the sheep. Wells *et al*. [24] chose the period of 1 min of sinusoidal change in walking speed between 3.2 and 6.4 km h^-1^ so as to emphasize the faster *V̇*_E_ response against such a locomotive exercise. As a result, the *A* and *PS* values of the *V̇*_E_ were tightly coupled to those of the *V̇*CO_2_. Moreover, the *PS* for the *V̇*_E_ even in the shortest period (1 min) was remarkably delayed from locomotive parameters [24]. Our present study examined various oscillation periods from 1 min to 10 min, and found that ventilatory and gas exchange responses were highly dependent on the oscillation periods in association with a different exercise mode (Fig 3). The locomotive parameters showed quite faster adaptation in response to sinusoidal changes in treadmill speed (Fig 4C), therefore, the locomotor dynamics were almost synchronized with treadmill speed oscillation and represented a faster response dissociated from the ventilatory and gas exchange dynamics. These results suggested that locomotive parameters were not a determinant factor for the speed of *V̇*_E_ response during SW.

A significantly greater *A* and smaller *PS* in the *V̇*_E_ compared to other variables at the period of 2 min during SW was the first observation (Fig 3A and 3E), because such a smaller *PS* and greater *A* during SC have not been reported yet [4–7]. This finding of our present study indicated a different control mechanism for the *V̇*_E_ between SW and SC derived from the specific trend of the *PS* accompanied by an increase in the sinusoidal oscillation during SW. At the specific period of 2 min during SW, an abrupt increase in the *V̇*_E_ adjustment occurred that is dissociated from both the metabolic potential and motor behavior. Because a significantly greater *PS* was observed for *V̇*_E_ than for *V̇*CO_2_. (Fig 3E and 3F, p < 0.05). With regard to disparate respiratory mechanisms, somatic afferents from working muscles activated the central respiratory neurons indirectly via a polysynaptic pathway from the spinal dorsal horn to the medullary ventral respiratory group through the lateral parabrachial nucleus [25].

It was interesting to note that the steeper slope of regression line of the *A* ratio of *V̇*_E_ as a function of *V̇*CO_2_ during SW was remarkably observed compared to that during SC (Fig 5A). Each slope of the regression line was 1.160 during SW and 0.804 during SC, indicating that the slope was 1.44 times steeper during SW than SC, even though the metabolic demand was equivalent between both exercises. At a given *V̇*CO_2_, if a slightly higher *V̇*_E_ would be stimulated by a specific exercise mode, then the central feed-forward command or upward information from afferent neural activity could be partly related to the additional augment in *V̇*_E_ during SW rather than the humoral outcome via the equivalent metabolic demand during SC [8–10]. The observed differences in the ventilatory response between walking and cycling would be attributed to the neuromuscular afferent flow into the medial brain and respiratory-locomotor generation center [26–28].

However, with respect to locomotion behavior, as shown in Fig 5D, the *A* ratios of *V̇*_E_ at all periods during SW were significantly related to the *A* ratios of the stride length, but not step frequency (Fig 5E). This must be because the gluteus maximus muscle would be strongly activated during SW. It can be speculated that this variation of muscle activation (i.e., motor unit alteration) provides stronger muscle afferent feedback to control ventilation during SW. On the other hand, the step frequency variation was not affected to the *A* ratios of *V̇*_E_ during SW (Fig 5E). This observation suggested that the adjustment in ventilation could be mainly attributed to the activation of locomotor muscles rather than the impact of step frequency in the transient phases of sinusoidal speed change during walking.

Changes in P_ET_CO_2_ during SW displayed small *A* oscillations ranging from 0.5 ± 0.1 mmHg to 2.1 ± 0.7 mmHg (Fig 3C), which were very similar to those during SC at all periods. This demonstrated that the strong metabolic feedback system via the PaCO_2_ that adjusts ventilation might work during SW as well as during SC, suggesting that such a feedback system is independent of the exercise mode. The absence of a significant correlation of the *A* ratio between *V̇*_E_ and P_ET_CO_2_ indicates that P_ET_CO_2_ did not tightly regulate ventilation during SW (Fig 5C). Instead, an alteration of P_ET_CO_2_ could reflect an error signal to the arterial and central chemoreceptor, which will be further related to *V̇*_E_. An important point needs to be clarified about PaCO_2_ homeostatics during exercise, that is, the ventilation in all types of exercise modes are related or proportional to the pulmonary gas exchange dynamics particularly at the transition phases. During sinusoidal change in work rate during SC, the ventilatory-gas exchange matching occurred to prevent an abrupt increase in PaCO_2_, which would be derived from an increase in *V̇*CO_2_, thereby minimizing the changes in PaCO_2_ at an abrupt change in the exercise intensity [29–34].

Carter *et al*. [14] demonstrated that the speed of *V̇*O_2_ dynamics at the step transition was not significantly different between running and cycling at moderate and severe exercise intensities. Under the equivalent metabolic demand during SW and SC in our study, accelerated *V̇*O_2_ dynamics during SW was demonstrated by the significant alterations in the *A* at 1 min and 5 min periods and in the *PS* at all periods except for 5 min period. The apparent difference of exercise mode even at the equivalent metabolic demand would be attributed to an increase in the slow-twitch fiber recruitment, being strongly related to the speed of the *V̇*O_2_ dynamics or oxidative capacity [3,7,15].

## Conclusions

Ventilatory and gas exchange dynamics against sinusoidal changes in work rate and/or treadmill speed at the equivalent metabolic demand clearly showed different dynamics depending on the oscillation periods, even though the locomotive parameters quickly followed such oscillations. Fundamentally, the *V̇*_E_ response during SW is linked to a signal related to the metabolic activation (i.e., *V̇*CO_2_) and stride length, but not step frequency. Interestingly, a 1.44 times steeper slope of regression line between the *V̇*_E_ and *V̇*CO_2_ was observed during SW than during SC. Judging from obtained results, such a different ventilatory adjustment between SW and SC would be derived from central command in proportion to locomotor muscle recruitment (feedforward) and muscle afferent feedback during SW even if the metabolic demand was equivalent between both exercises.

## Supporting Information

S1 Fig The *PS* between V_E_, VO_2_, VCO_2_, and HR and the treadmill speed sinusoidal oscillations (SW, A panel), the work rate sinusoidal oscillations (SC, B panel) all oscillation periods from 1 min to 10 min. The cardiodynamic hypothesis could be accepted by the rational dynamics of these variables during SC and SW except 2 min period of SW. Interestingly, only at the period of 2 min during SW, the *PS* of _E_ was the smallest compared with other variables. * p < 0.05 vs. V_E_, ° p < 0.05 vs. VCO_2_, and □ p < 0.05 vs. VO_2_.
(TIF)

S1 Table Amplitudes of gas exchange variables during sinusoidal walking and sinusoidal cycling. Breath-by-breath ventilation (*V̇*_E_, BTPS), O_2_ uptake (*V̇*O_2_, STPD), CO_2_ output (*V̇*CO_2_, STPD), and heart rate (HR) were determined. Data are shown by mean ± SE.
(TIF)

S2 Table Phase shifts of gas exchange variables during sinusoidal walking and sinusoidal cycling. Breath-by-breath ventilation (*V̇*_E_, BTPS), O_2_ uptake (*V̇*O_2_, STPD), CO_2_ output (*V̇*CO_2_, STPD), and heart rate (HR) were determined. Data are shown by mean ± SE.
(TIF)
