# Supplementary figures and images for: Dynamic Characteristics of Ventilatory and Gas Exchange during Sinusoidal Walking in Humans
Source: PLoS One. 2017 Jan 11;12(1):e0168517. doi: 10.1371/journal.pone.0168517 (PMC5226792; doi:10.1371/journal.pone.0168517)

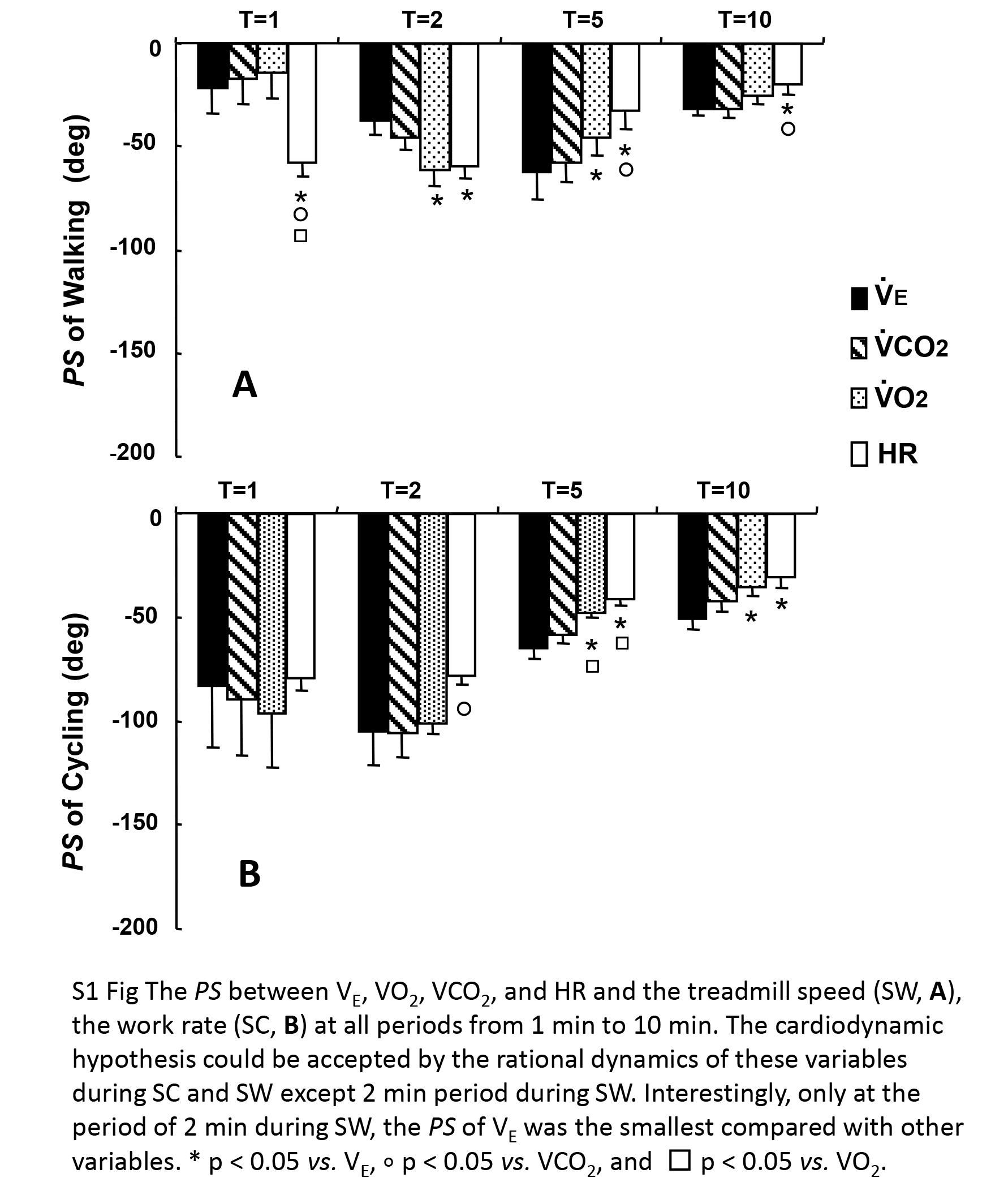

Supplement: S1 Fig — The cardiodynamic hypothesis could be accepted by the rational dynamics of these variables during SC and SW except 2 min period of SW. Interestingly, only at the period of 2 min during SW, the PS of E was the smallest compared with other variables. * p < 0.05 vs. VE, ° p < 0.05 vs. VCO2, and □ p < 0.05 vs. VO2. (TIF) [file pone.0168517.s001.tif]

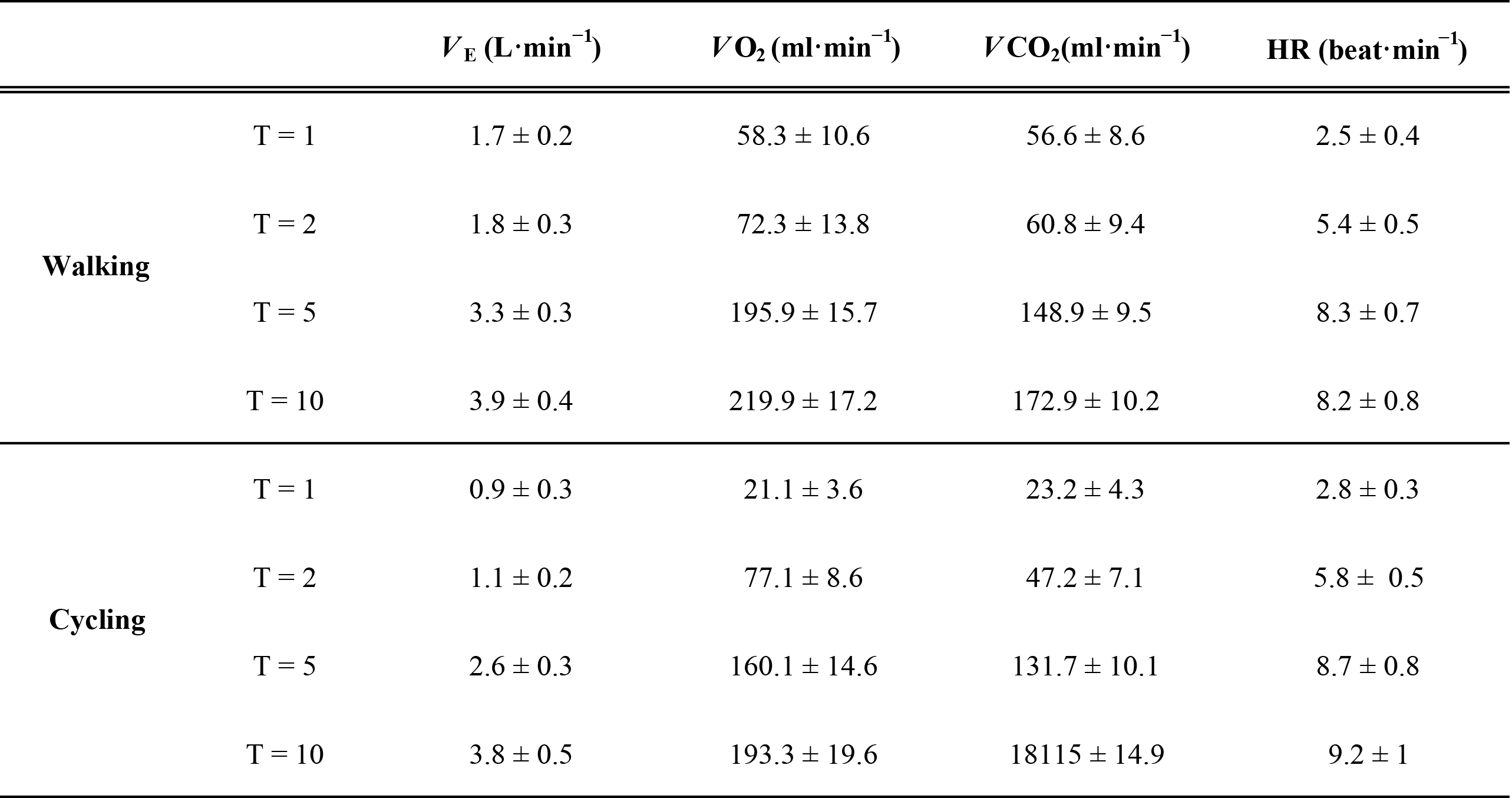

Supplement: S1 Table — Breath-by-breath ventilation (V̇E, BTPS), O2 uptake (V̇O2, STPD), CO2 output (V̇CO2, STPD), and heart rate (HR) were determined. Data are shown by mean ± SE. (TIF) [file pone.0168517.s002.tif]

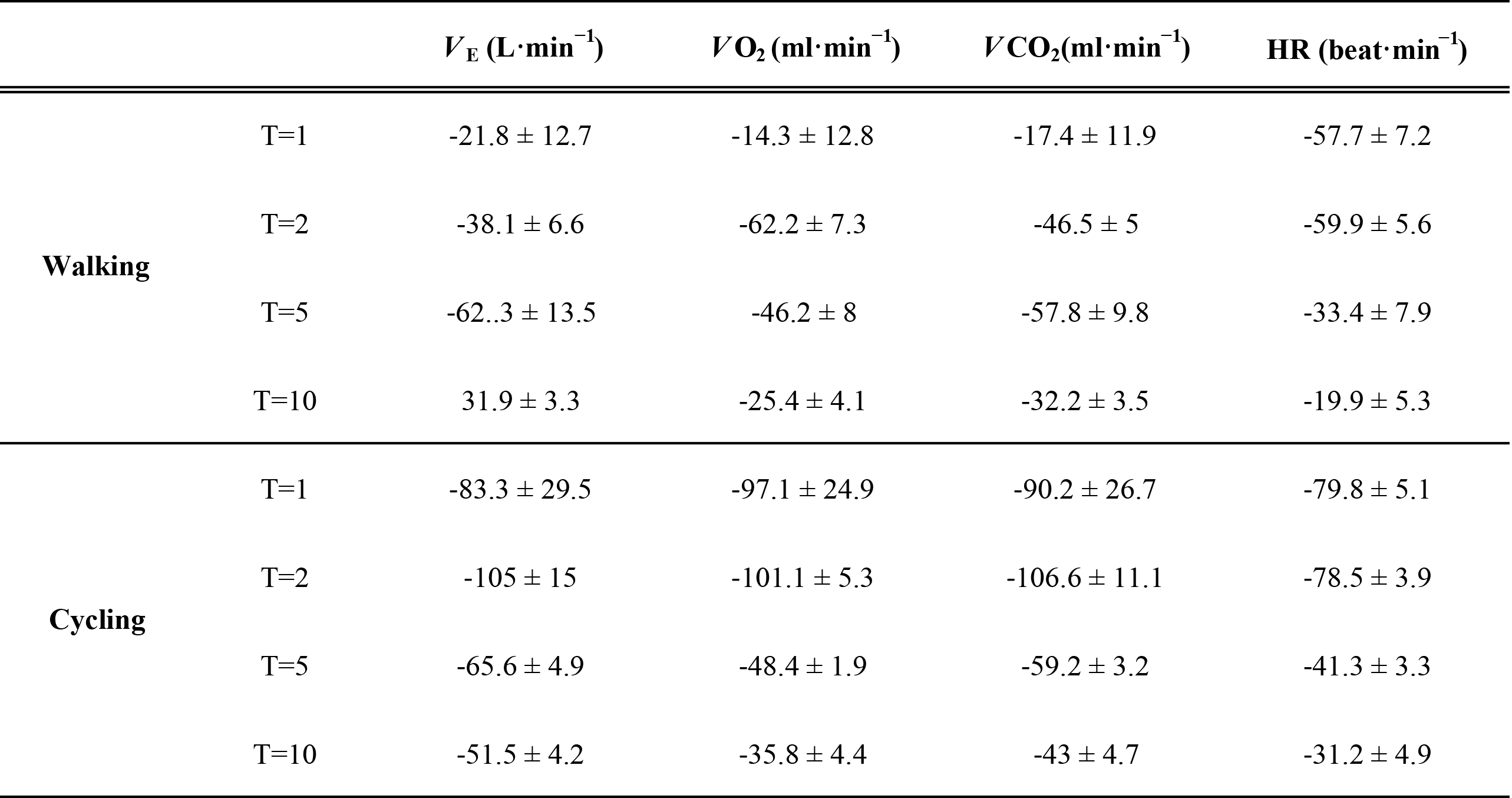

Supplement: S2 Table — Breath-by-breath ventilation (V̇E, BTPS), O2 uptake (V̇O2, STPD), CO2 output (V̇CO2, STPD), and heart rate (HR) were determined. Data are shown by mean ± SE. (TIF) [file pone.0168517.s003.tif]
